# Veterinary Medical Associaion of New Jersey

**Published:** 1897-05

**Authors:** S. Lockwood

**Affiliations:** Secretary


### VETERINARY MEDICAL ASSOCIATION OF NEW JERSEY.

THE thirteenth annual meeting was held at the Continental Hotel, Newark, N. J., on April 8, 1897. Meeting called to order at 11.30 A.M., President Hawk in the chair. There were but fifteen members present. After the roll-call, the minutes of the previous regular meeting were read and approved, also the minutes of a special meeting held on December 22, 1896.

The Secretary's report showed a steady growth in numbers, and the Treasurer's report a rapid increase financially. There was but one applicant for membership present—Dr. F. A. Zucker, of Elizabeth—who was unanimously elected after being examined by the Board of Censors.

The election of officers resulted in the unanimous election of President, Dr. W. H. Arrowsmith; First Vice-President, Dr. R. C. Vail; Second Vice-President, Dr. W. Runge; Secretary, Dr. S. Lockwood; Treasurer, Dr. B. F. King; Trustees, Drs. W. Runge, J. W. Hawk, A. W. Oxford, W. Gall, and B. F. King, all being elected for two years.

The essayists being absent, Dr. Arrowsmith gave a description of an operation which he had performed—a laryngotomy for the removal of an osseous growth, which caused roaring, and he exhibited the electric lamp and instruments used. The operation was performed at his hospital in Jersey City, and was successful.

The President, in his address to the Association, emphasized the necessity of each member trying to improve the meetings by being more prompt at the time appointed, reporting interesting cases, inviting others of the profession to join the Association, and, when the Association is trying to get new laws passed, not to leave all the work for the committee, but each one see the member from his district and explain the need of such a measure; then when he sees the bill he will take an interest in it. Adjourned at 1.15 for dinner.

Reconvened at 2.45 o'clock. Dr. Dustan, chairman of the Legislative Committee, made a report of the work of the committee and of the defeat of the bill, after going to third reading in the House. The bill was the same as the New York and Pennsylvania bills providing for a State Board of Veterinary Examiners. The report was accepted and the committee discharged. The Secretary read a letter from the Pasteur Monument Committee. Dr. Vail told of the action taken by the Pennsylvania Association, and moved that we give a like amount; motion lost. Moved and carried to pass a subscription paper, and let each contribute what sum he desired. The Secretary was instructed to write each member who was not present, giving all an opportunity to contribute.

The President appointed as delegates to the New York State and County Associations Drs. W. Runge, R. C. Vail, and J. W. Hawk; to the Pennsylvania Association Drs. F. A. Zucker, J. C. Dustan, and William Gall; essayists for next meeting, Drs. William Gall, W. Runge, A. D. Edwards, and B. F. King.

Moved and carried to meet at Trenton in October. Adjourned at 5 P.M.

S. LOCKWOOD,
Secretary.

